# Root-Specific Expression of *Vitis vinifera* VviNPF2.2 Modulates Shoot Anion Concentration in Transgenic *Arabidopsis*

**DOI:** 10.3389/fpls.2022.863971

**Published:** 2022-05-25

**Authors:** Yue Wu, Sam W. Henderson, Rob R. Walker, Matthew Gilliham

**Affiliations:** ^1^Australian Research Council (ARC) Centre of Excellence in Plant Energy Biology, School of Agriculture, Food and Wine and Waite Research Institute, University of Adelaide, Glen Osmond, SA, Australia; ^2^School of Biomedicine, University of Adelaide, Adelaide, SA, Australia; ^3^Commonwealth Scientific and Industrial Research Organisation (CSIRO), Glen Osmond, SA, Australia; ^4^Australian Research Council (ARC) Industrial Transformation Training Centre for Innovative Wine Production, School of Agriculture, Food and Wine and Waite Research Institute, University of Adelaide, Glen Osmond, SA, Australia

**Keywords:** salinity, chloride exclusion, grapevine, NPF, nitrate

## Abstract

Grapevines (*Vitis vinifera* L., *Vvi*) on their roots are generally sensitive to salt-forming ions, particularly chloride (Cl^–^) when grown in saline environments. Grafting *V. vinifera* scions to Cl^–^-excluding hybrid rootstocks reduces the impact of salinity. Molecular components underlying Cl^–^-exclusion in *Vitis* species remain largely unknown, however, various anion channels and transporters represent good candidates for controlling this trait. Here, two nitrate/peptide transporter family (NPF) members *VviNPF2.1* and *VviNPF2.2* were isolated. Both highly homologous proteins localized to the plasma membrane of Arabidopsis (*Arabidopsis thaliana*) protoplasts. Both were expressed primarily in grapevine roots and leaves and were more abundant in a Cl^–^-excluding rootstock compared to a Cl^–^-includer. Quantitative PCR of grapevine roots revealed that *VviNPF2*.*1* and *2.2* expression was downregulated by high [NO_3_^–^] resupply post-starvation, but not affected by 25 mM Cl^–^. VviNPF2.2 was functionally characterized using an Arabidopsis enhancer trap line as a heterologous host which enabled cell-type-specific expression. Constitutive expression of *VviNPF2.2* exclusively in the root epidermis and cortex reduced shoot [Cl^–^] after a 75 mM NaCl treatment. Higher expression levels of *VviNPF2.2* correlated with reduced Arabidopsis xylem sap [NO_3_^–^] when not salt stressed. We propose that when expressed in the root epidermis and cortex, VviNPF2.2 could function in passive anion efflux from root cells, which reduces the symplasmic Cl^–^ available for root-to-shoot translocation. VviNPF2.2, through its role in the root epidermis and cortex, could, therefore, be beneficial to plants under salt stress by reducing net shoot Cl^–^ accumulation.

## Introduction

Salinity is a major challenge for salt-sensitive crops (Walker et al., 2002a; Munns and Gilliham, 2015). Osmotic stress, caused by high dissolved salt concentrations in the root zone, occurs rapidly and reduces tissue growth. Accumulation of sodium (Na^+^) and Cl^–^ ions within cells affects metabolic processes, which can lead to toxicity and cell death (Munns et al., 2020; Van Zelm et al., 2020). Plants possess two main mechanisms for tolerating salinity. Osmotic stress-tolerant plants are more effective in maintaining stomatal movements and leaf expansion compared to sensitive plants (Munns, 2011). Ionic stress-tolerant plants compartmentalise ions into vacuoles of specific cell types to minimise ionic effects on metabolism and to contribute toward exclusion of Na^+^ and Cl^–^ from key organs such as laminae (Rajendran et al., 2009; Isayenkov and Maathuis, 2019; Munns et al., 2020).

Grapevines (*Vitis vinifera* L.) are cultivated for table, dried, and wine grape production. They are moderately sensitive to salinity (Maas and Hoffman, 1977; Zhou-Tsang et al., 2021), which can affect both grape and wine production. Significant uptake of salt-forming ions by grapevine roots, and their transfer to the shoot, may cause leaf burn and affects berry development (Walker, 1994), which can reduce both crop yield and quality (Prior et al., 1992a,b; Walker et al., 2002b,^2019^; Stevens et al., 2011; Baby et al., 2016). Release of accumulated Na^+^ and Cl^–^ from fruit during crushing may lead to an adverse effect on fermentation (Berg and Keefer, 1958; Donkin et al., 2010; Li et al., 2013) and potential unfavourable sensory properties within wine (Walker et al., 2003; De Loryn et al., 2014). Exceeding the legal requirements for Na^+^ and Cl^–^ concentrations within wine (Leske et al., 1997; De Loryn et al., 2014) makes wine unsaleable. Grafting salt-sensitive *V. vinifera* scions to salt-excluding *Vitis* spp. rootstocks protects vines and berries from salinity by limiting the amount of Na^+^ and Cl^–^ translocated from root to shoot (Zhou-Tsang et al., 2021). The Na^+^ exclusion mechanism is governed by Na^+^-selective high-affinity potassium (K^+^) transporters (HKT) expressed in the root vasculature (Henderson et al., 2018; Wu et al., 2020). By contrast, the genes controlling Cl^–^-exclusion remain largely unknown. Cl^–^-exclusion could be achieved through several mechanisms including efflux from the root (Abbaspour, 2008; Abbaspour et al., 2013), vacuolar sequestration (Teakle and Tyerman, 2010; Walker et al., 2018), reduced xylem loading in the root stele (Tregeagle et al., 2006; Gong et al., 2011), and increased retrieval from xylem sap to xylem parenchyma (Colmenero-Flores et al., 2007; Teakle and Tyerman, 2010). Few Cl^–^-permeable membrane proteins contributing to these processes have been discovered in plants (reviewed by Li et al., 2017b; Wege et al., 2017), and they are often selective for both nitrate (NO_3_^–^) and Cl^–^.

The main pathway for Cl^–^ uptake is the secondary active 2H^+^/Cl^–^ symporter (Sanders, 1980; Felle, 1994), which drives proton-coupled Cl^–^ influx. In maize (*Zea mays*), the 2H^+^/Cl^–^ symporter belongs to the NPF6 clade of the Nitrate Transporter 1/Peptide Transporter Family (NPF) and is encoded by *ZmNPF6.4*, which was permeable to both Cl^–^ and NO_3_^–^ at acidic pH in *Xenopus laevis* oocytes (Wen et al., 2017). The *Arabidopsis thaliana* ortholog, AtNPF6.3, was first characterized as a 2H^+^/NO_3_^–^ symporter (Liu et al., 1999), however, it contributed to Cl^–^-induced salt toxicity when NO_3_^–^ was absent (replaced with NH_4_^+^), suggesting that AtNPF6.3 cotransports Cl^–^ as well as NO_3_^–^ in plants (Liu et al., 2020). These properties are like MtNPF6.5 from *Medicago truncatula*, which transported both Cl^–^ and NO_3_^–^, but was Cl^–^ selective in oocytes (Xiao et al., 2021). Another class of NPF proteins (NPF2) functions in passive anion efflux from roots. AtNPF2.7 functioned in NO_3_^–^ (but not Cl^–^) efflux from root cortical cells under acid load (Segonzac et al., 2007). Its homolog, AtNPF2.3, contributed toward passive NO_3_^–^ loading to xylem vessels from pericycle cells under salt stress (Taochy et al., 2015). AtNPF2.4 was more permeable to Cl^–^ than NO_3_^–^ in *X. laevis* oocytes and was proposed to facilitate root-to-shoot Cl^–^ transfer (Li et al., 2016). Finally, *AtNPF2.5* downregulation in the Arabidopsis root cortex correlated with shoot Cl^–^ accumulation, suggesting that it effluxes Cl^–^ to the outer medium (Li et al., 2017a). Collectively, these studies demonstrate that NPF proteins play crucial roles in net plant Cl^–^ uptake and are excellent candidates that might control the shoot Cl^–^-exclusion trait in grapevines.

Gong et al. (2011) screened the progeny from a cross between the Cl^–^-excluding grapevine rootstock 140 Ruggeri and the Cl^–^-includer K51-40. The shoot Cl^–^ concentration of the progeny showed no clear segregation, suggesting that the Cl^–^ exclusion trait was controlled by more than one gene in that population. Comparative microarray analysis of gene expression between the roots of 140 Ruggeri and K51-40 identified two putative anion transporters from the NPF Family, *VviNPF2.1* and *VviNPF2.2*, that were both significantly more abundant in the roots of the Cl^–^-excluder 140 Ruggeri compared to the Cl^–^-includer K51-40 when differences in laminae [Cl^–^] were apparent (Henderson et al., 2014; Supplementary Table 1). *VviNPF2.1* and *VviNPF2.2* were, therefore, proposed as candidate genes that may contribute to the Cl^–^ exclusion trait, but their functions remained unknown. Here, VviNPF2.1 and VviNPF2.2 were isolated and functionally investigated to determine their involvement in grapevine Cl^–^ exclusion.

## Materials and Methods

### Gene Cloning

The coding sequences (CDS) of *VviNPF2.1* (VIT_06s0004g03520) and *VviNPF2.2* (VIT_06s0004g03530) and their respective promoters (1.2–1.6 kb upstream of the start codon of CDS) were amplified from *V. vinifera* (cv. Cabernet Sauvignon) root cDNA with Phusion High-Fidelity DNA Polymerase (New England Biolabs, Ipswich, MA, United States), using the primers in Supplementary Table 2. The cloned promoter region of *VviNPF2.1* is named *proVviNPF2.1* (−1,206 to −1 bp), and the promoter of *VviNPF2.2* is named *proVviNPF2.2* (−1,551 to −1 bp) in this study. The PCR products were ligated into the entry vector pCR8 using the pCR8/GW/TOPO TA Cloning Kit (Invitrogen, Waltham, MA, United States) or the vector pENTR using the pENTR/D-TOPO Cloning Kit (Invitrogen, Waltham, MA, United States) as per the manufacturer’s instructions. One Shot TOP10 *Escherichia coli* (Invitrogen, Waltham, MA, United States) were transformed with the entry vectors as per the manufacturer’s instructions. Plasmids were harvested using the ISOLATE II Plasmid Mini Kit (Bioline, London, United Kingdom), and successful cloning was confirmed by Sanger sequencing.

### Subcellular Localization in Arabidopsis Mesophyll Protoplasts

The *VviNPF2.1 and VviNPF2.2* CDS in pCR8 vectors were recombined into both pYFP-attR and pattR-YFP using LR Clonase II (Life Technologies, Carlsbad, CA, United States) to generate vectors encoding 35S:*EYFP-VviNPF* and 35S:*VviNPF-EYFP*, respectively. The vectors generated by LR recombination were used to transform *Escherichia coli* DH5α competent cells and plasmids were harvested.

*A. thaliana* mesophyll protoplasts were harvested by the Tape-Arabidopsis Sandwich method (Wu et al., 2009). The protoplasts were transfected using a modified TEAMP method (Yoo et al., 2007). Approximately 15 μg of each of the recombinant plasmids were added to 0.2 ml of MMg solution (4 mM MES, 0.4 M D-mannitol, 15 mM MgCl_2_) containing approximately 5 × 10^4^ protoplasts at room temperature. An equal volume of 30% (w/v) polyethylene glycol (PEG, molecular weight 4,000) solution in 0.1 M CaCl_2_ and 0.2 M D-mannitol was added to the mixture and incubated at room temperature for 5 min. W2 wash solution (1 M MES, 0.4 M D-mannitol, 15 mM KCl, 10 mM CaCl_2_, and 5 mM MgCl_2_) was slowly added to the mixture to a total volume of 2 ml after incubation. The mixture was gently mixed and the protoplasts were pelleted by centrifugation at 100 × *g* for 1 min. The supernatant was discarded and the wash step was repeated twice using a W2 solution. The protoplasts were resuspended with 1 ml of W2 solution and transferred to a 12-well plate pre-coated with 1% BSA for incubation. The protoplasts were incubated under a normal daylight regime for 16 h at room temperature. The transfected protoplasts were imaged after incubation using a Nikon A1R confocal laser-scanning microscope and NIS-Elements C software (Nikon Corporation, Minato, Tokyo, Japan). FM4-64 was added to the protoplast mixture in a 1 in 1,000 ratio as a plasma membrane (PM) marker, and the protoplasts were imaged after a 10–15-min incubation at room temperature. YFP was imaged using a FITC filter (500–550 nm), 488 nm excitation wavelength, 525 nm emission wavelength; FM4-64 was imaged using a TRITC filter (570–620 nm), 561.1 nm excitation wavelength, 595 nm emission wavelength; chlorophyll was imaged using a Cy5 filter (650–720 nm), 640.4 nm excitation wavelength, 700 nm emission wavelength. YFP and FM4-64 signals were detected separately in channel mode.

### RT-qPCR

Two sets of grapevine hydroponically grown rooted leaf cDNA or RNA samples were obtained from Henderson et al. (2014). The cDNA samples of stellar-enriched and cortex-enriched 25 mM Cl^–^ treated grapevine roots were obtained for qPCR gene expression analysis. The RNA samples of grapevine whole roots treated with control or 25 mM Cl^–^ solutions were used to make cDNA for qPCR as described by Wu et al. (2020).

The cDNA samples of the Cabernet Sauvignon hardwood cuttings in the study by Wu et al. (2020) were used for qPCR. Hardwood cuttings with 4–6 nodes were collected before winter pruning and propagated. The cDNA series consists of grapevine leaves at the growth stage when 5 leaves are separated (E-L stage 12) (Dry et al., 2004), young inflorescences (E-L stage 12), well-developed inflorescences (E-L stage 17), roots at E-L stage 26, mature leaves and petiole samples (E-L stage 27), pea-sized green berries (E-L stage 31), and berries post-veraison (E-L stage 36–37) as described by Wu et al. (2020).

The root cDNA samples of the grapevine green cuttings after NO_3_^–^ treatments in the study by Wu et al. (2020) were used for qPCR. Grapevine green cuttings with 2 nodes and mature leaves were obtained from glasshouse-grown, potted vines of Cabernet Sauvignon, 140 Ruggeri and K51-40, for the NO_3_^–^ responses experiment. Rooted cuttings were starved with 0.8 mM NO_3_^–^ for 2 weeks and then supplied with nutrient solutions containing 0.8 or 12 mM NO_3_^–^. Root samples were taken 24 h after the NO_3_^–^ treatments were applied, and were immediately frozen in liquid nitrogen for total RNA extraction and cDNA synthesis as described by Wu et al. (2020).

qPCR primers specific to *VviNPF2.1* and *VviNPF2.2* were designed to amplify fragments between 80 and 250 bp (Supplementary Table 2). The qPCR primers of the 3 housekeeping genes, α-Tubulin (*VviTUA*), Ubiquitin-conjugating-enzyme-like (*VviUBC*), and Elongation-factor-1-α (*VviEF1a*), were obtained from Wu et al. (2020). qPCR was performed using QuantStudio 12K Flex Real-Time PCR System (Life Technologies, Carlsbad, CA, United States) and KAPA SYBR FAST Universal qPCR kit (KAPA Biosystems, Cape Town, South Africa). Standard curve qPCR was performed to obtain the reaction efficiency of each primer pair. PCR fragments of each gene were amplified from the grapevine cDNA using the above-mentioned primers and *Taq* DNA Polymerase (New England Biolabs, Ipswich, MA, United States). The fragment sizes of PCR products were checked by agarose gel and the PCR products were purified and sequenced to confirm primer specificity.

For qPCR using grapevine cDNA samples, PCR fragments with correct sequences were diluted to 10^11^ copies/μl, and then 1 in 8 serially diluted for use as standard curve templates for qPCR. qPCR and standard curve PCR were performed on a QuantStudio 12K Flex Real-Time PCR System (Life Technologies, Carlsbad, CA, United States). Each qPCR reaction was performed in triplicate. Each 10 μl reaction consisted of 1X KAPA SYBR FAST Universal mix (KAPA Biosystems, Cape Town, South Africa), 1X ROX Low, 250 nM forward and reverse primers, and 1 μl of 1 in 10 diluted cDNA. The qPCR consisted of 40 cycles of a 2-step protocol: 95°C 3 s, 56°C 20 s (followed by data acquisition). Standard curves were generated by the QuantStudio 12K Flex Real-Time PCR System v1.2.2 (Life Technologies, Carlsbad, CA, United States), which also calculated the reaction efficiency of each primer pair. Expression levels (E) of *VviNPF2.1* and *VviNPF2.2* were calculated relative to sample 1 of each experiment (as described in the figure legends) using the Equation E = (2*efficiency)^(CT_sample_–CT_sample 1_). Expression levels were normalized to the geometric mean of the expression levels of the 3 housekeeping genes (Vandesompele et al., 2002).

### Gene Expression in *Xenopus* Oocytes

The *VviNPF2.1* and *VviNPF2.2* CDS in pCR8 vector were recombined into the *Xenopus laevis* oocyte expression vector pGEMHE-DEST (Shelden et al., 2009) using LR Clonase II (Life Technologies, Carlsbad, CA, United States) to generate vectors encoding T7*:VviNPF*. The *pGEMHE* recombinant vectors were linearized with *Sbf*I or *Nhe*I (New England Biolabs, Ipswich, MA, United States). The capped RNA (cRNA) for oocyte expression was synthesized with the mMESSAGE mMACHINE T7 Transcription Kit (Invitrogen, Waltham, MA, United States) using the linearized vectors as templates.

Stages IV and V *X. laevis* oocytes were selected and were injected with 25 ng of *VviNPF2.1* or *VviNPF2.2* cRNA, or 42 nl of water. The oocytes were incubated in a Ca^2+^ Ringer’s solution [96 mM NaCl, 2 mM KCl, 5 mM MgCl_2_, 5 mM HEPES, 0.6 mM CaCl_2_, 5% w/v horse serum, 500 μg ml^–1^ tetracycline and 1x penicillin–streptomycin (Sigma P4333)] for 2 days post-injection.

### Anion Tracer Fluxes and [Cl^–^] Measurements in *Xenopus* Oocytes

*Torpedo marmorata* CLC-0, a chloride channel with known Cl^–^ and NO_3_^–^ permeability (Bergsdorf et al., 2009), was used as a positive control. For the Cl^–^ tracer influx assays, the influx buffer was made by adding 13.3 μl of H^36^Cl stock solution (11.3 mg/ml Cl^–^, 75 μCi/ml) into 1 ml of ND96 buffer (96 mM NaCl, 2 mM KCl, 1.8 mM CaCl_2_, 1 mM MgCl_2_ and 5 mM HEPES, pH 7.4). Water-injected and gene-expressing oocytes were incubated in the influx buffer for 1 h. The oocytes were taken out of the efflux buffer and washed three times in ice-cold ND96 buffer, then each oocyte was transferred to a scintillation vial containing 200 μl of 10% (w/v) SDS solution. For Cl^–^ efflux assays, 42 nl of H^36^Cl stock solution (11.3 mg/ml Cl^–^, 75 μCi/ml) was injected into each of the water-injected and gene-expressing oocytes. The control group oocytes were immediately washed three times in ice-cold ND96 buffer, then each oocyte was transferred to a scintillation vial containing 200 μl of 10% (w/v) SDS solution. The efflux group oocytes were quickly transferred to room temperature Cl^–^-free ND96 buffer (96 mM Na gluconate, 2 mM K gluconate, 1.8 mM Ca gluconate, 1 mM Mg gluconate, and 5 mM HEPES, pH 7.4) to allow Cl^–^ efflux for 1 h. The oocytes were taken out of the efflux buffer and washed three times in ice-cold Cl^–^-free ND96 buffer, then each oocyte was transferred to a scintillation vial containing SDS solution. All oocytes were allowed to dissolve in the SDS overnight, then 4 ml of liquid scintillation cocktail was added to each vial. The vials were loaded onto a LS6500 multi-purpose scintillation counter (Beckman Coulter, Brea, CA, United States) and energy emission was counted for 2 min in cpm (counts per min) with the discriminators set to 200–800 KeV.

For the NO_3_^–^ tracer influx experiment, the influx buffer was made by adding 30 mM of Na^15^NO_3_ (99.3% atom) into the ND96 buffer (pH 7.4). Water-injected and gene-expressing oocytes were incubated in the influx buffer for 2 h. The oocytes were taken out of the efflux buffer and washed three times in ice-cold ND96 containing 30 mM NaNO_3_, then they were transferred into tin capsules in a 96-well-plate (2 oocytes per capsule). For the NO_3_^–^ efflux experiment, 42 nl of 300 mM K^15^NO_3_ (99.3% atom) was injected into each of the water-injected and gene-expressing oocytes. The oocytes were immediately transferred into an ND96 buffer (96 mM NaCl, 2 mM KCl, 1.8 mM CaCl_2_, 1 mM MgCl_2_, and 5 mM MES, pH 5.5) to allow efflux for 1 h. The oocytes were then washed three times in ice-cold ND96 buffer (pH 5.5), followed by transfer into tin capsules (3 oocytes per capsule). The tin capsules were oven-dried at 50°C for 3 days. The samples were sent for analysis in a stable isotope ratio mass spectrometer Nu Horizon IRMS (Nu Instruments, Wrexham, United Kingdom) in the University of Adelaide Stable Isotope Facility for δ^15^ N.

For the oocyte nominal [Cl^–^] tests, cRNA or water-injected oocytes were incubated in the Ca^2+^ Ringer’s solution for 2 days. The oocytes were washed in ice-cold HMg solution (6 mM Mg gluconate, 1.8 mM Ca gluconate, 10 mM MES, pH 6.5) and digested in 550 μl of 1% HNO_3_ in 1.5 ml tubes (7–10 oocytes per tube). The [Cl^–^] in the supernatant was tested using a Sherwood Model 926 Chloride Analyzer (Sherwood Scientific, Cambridge, United Kingdom) as per the manufacturer’s protocol, and the [Cl^–^] concentration in the solution was converted to [Cl^–^] per oocyte assuming an average oocyte is 400 nl in volume.

### Root Epidermis and Cortex-Specific Expression of *VviNPF2.2* in an Arabidopsis Enhancer Trap Line

The Arabidopsis enhancer trap line J1551 (C24 ecotype background) for root epidermis and cortex-specific transgene expression was obtained from Plett et al. (2010). The *VviNPF2.1* and *VviNPF2.2* CDS in the entry vectors were recombined into the pTOOL5-*UAS_*GAL*4_* destination vector (obtained from Plett, 2008), respectively, using LR Clonase II (Life Technologies, Carlsbad, CA, United States) to generate the binary vectors encoding *UAS_*GAL*4_*:*VviNPF2.1* and *UAS_*GAL*4_*:*VviNPF2.2*. In these constructs, the full-length NPF genes are driven by the upstream activation sequence (UAS) which is induced by GAL4-VP16. When introduced into the Arabidopsis enhancer trap lines, the NFP genes will be *trans*-activated in the same cell types as the marker mGFP5-ER (Haseloff, 1998). The binary vector was used to transform *A. tumefaciens* strain Agl-1 using the freeze-thaw method. The Arabidopsis enhancer trap J1551 plants were transformed using the Agrobacterium-mediated floral dip method (Clough and Bent, 1998). Transgenic lines of Arabidopsis were selected by the application of foliar spray of 120 mg/L Basta (Bayer Crop Science, Monheim am Rhein, Germany) mixed with 500 μl/L Silwet L-77 (plantMedia.com). The presence of T-DNA was confirmed by PCR.

Two heterozygous T2 lines of J1551 *UAS_*GAL*4_*:*VviNPF2.2*, lines 3 and 4 were selected by Basta foliar spray and genotyped using the root cDNA. The root epidermis and cortex-specific gene expression was confirmed by imaging the mGFP5-ER in roots of 4-week-old plants using a Nikon A1R confocal laser-scanning microscope (excitation/emission is 488 nm/500–550 nm). Homozygous T3 generation of lines 3 and 4 were also propagated and confirmed by Basta spray and subsequent fluorescence imaging of 4-week-old plants.

### Anion Concentration Measurements of Arabidopsis Lines Expressing *VviNPF2.2*

The Arabidopsis J1551 *UAS_*GAL*4_*:*VviNPF2.2* lines 3 and 4 were germinated and grown in the hydroponic system in the germination solution (GM) for 3 weeks and transferred into the standard basal nutrient solution (BNS) for 2 weeks as described by Conn et al. (2013).

For shoot anion tests post-salt stress, T2 *VviNPF2.2* expression lines were propagated hydroponically as described above. The BNS was then replaced with the high Na^+^ nutrient solution containing 75 mM NaCl (Conn et al., 2013). Five days post 75 mM NaCl treatment, the rosettes were harvested and the fresh weights were recorded. Each rosette was put into a 50-ml tube and for every 20 mg of rosette fresh weight, 1 ml of water was added into the tube. The rosettes and the liquid in the tubes were frozen at −20°C, thawed at room temperature and the tubes were vortexed; this process was repeated three times to fully release the cellular contents, and the resulting liquid samples were used for ion concentration measurements. The plant roots were harvested for RNA extraction and cDNA synthesis, and the cDNA was qPCR tested to differentiate the null segregants and *VviNPF2.2*-expressing individuals. Each qPCR reaction was performed in duplicate. The expression levels (E) of *VviNPF2.2* were calculated relative to the housekeeping gene *AtActin2* (At3G18780, qPCR primers as listed in Jha et al., 2010) and normalized to sample 1 using the Equation E = 2^–ΔΔCT^. Semi-qPCR was performed using several root cDNA samples of each line to visualize the relative expression levels on the gel. Samples 1–3 of each line and a non-transformed J1551 root cDNA sample were used as templates in two-step PCR reactions for 32 cycles. The PCR products were run on an electrophoresis gel and the gel image was taken using a ChemiDoc Touch Imaging System (Bio-Rad Laboratories, Hercules, CA, United States). Band intensities of the gel image were analyzed using Fiji (ImageJ) (Schindelin et al., 2012) and the intensities of *VviNPF2.2* bands were normalized to those of *AtAct2*. Standard qPCR was also performed to confirm the *VviNPF2.2* expression levels in lines 3 and 4 on a QuantStudio 12K Flex Real-Time PCR System (Life Technologies, Carlsbad, CA, United States). Each qPCR reaction was performed in duplicate. Each 10 μl reaction consisted of 1X KAPA SYBR FAST Universal mix (KAPA Biosystems, Cape Town, South Africa), 1X ROX Low, 250 nM forward and reverse primers, and 2 μl of 1 in 8 diluted Arabidopsis root cDNA. The qPCR consisted of 40 cycles of a 2-step protocol: 95°C 3 s, 57°C 20 s (followed by data acquisition). Expression levels (E) of *VviNPF2.2* were calculated relative to *AtActin2* using the Equation E = 2^[*VviNPF2.2*-CT_sample_ – *AtActin2*-CT_sample_ – (*VviNPF2.2*-CT_line 3 sample 1_ – *AtActin2*-CT_line 3 sample 1_)].

For Arabidopsis xylem sap [NO_3_^–^] analysis, the hydroponically grown 5-week-old T3 plants and non-transformed J1551 control plants were transferred into fresh BNS solution. For sap collection, the rosette was removed using a sharp razor blade, and the sap was collected for 30 min using fine pipette tips. The sap samples were then 1 in 20 diluted for NO_3_^–^ measurements.

The Cl^–^ concentrations of the liquid samples were measured using the Sherwood Model 926s Chloride Analyzer (Sherwood Scientific, Cambridge, United Kingdom). The NO_3_^–^ concentrations were measured using the reaction of NO_3_^–^ with salicylic acid under alkaline conditions as described by Cataldo et al. (1975). In a well of a flat bottom 96-well-plate, 3 μl of each sample was combined with 12 μl of H_2_SO_4_ containing 5% (w/v) salicylic acid and incubated at room temperature for 20 min. Then, 285 μl of 2N NaOH was mixed into each well and the absorbance at 410 nm (OD_410_) was measured. A series of KNO_3_ solutions from 0 to 10 mM were used for a standard curve and the [NO_3_^–^] of the samples was calculated using the standard curve.

### Statistical Analyses

Statistical analyses were performed using GraphPad PRISM v.7.00 for Windows (GraphPad Software, San Diego, CA, United States). All data are presented as mean ± SE. The means were compared using Student’s *t*-test or one-way ANOVA with Tukey’s multiple comparisons test.

## Results

### VviNPF2.1 and VviNPF2.2 Display High-Sequence Homology

**FIGURE 1:**
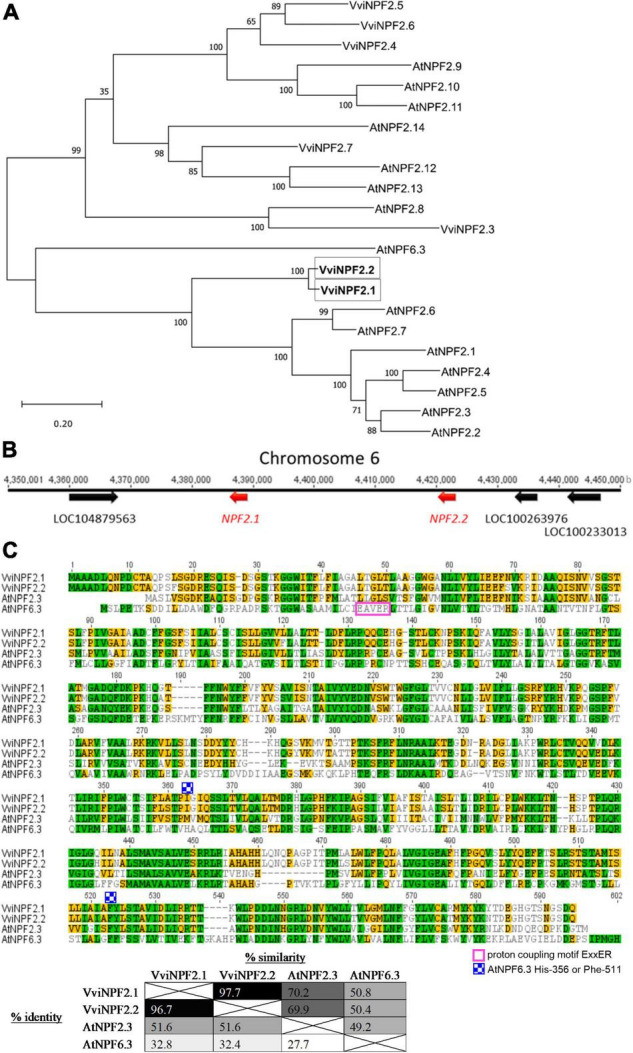
VviNPF2.1 and VviNPF2.2 share high-sequence homology. **(A)** Phylogenetic relationships of grapevine and Arabidopsis NPF2 family members, with AtNPF6.3 shown as the outgroup. The *AtNPF* and *VviNPF* gene sequences were obtained from the *A. thaliana* Col-0 reference genome (The Arabidopsis Genome Initiative, 2000) and the *V. vinifera* PN40024 genome database (Jaillon et al., 2007), respectively, using gene IDs listed in Léran et al. (2014). Protein alignments were generated using Clustal Omega, and a Maximum Likelihood phylogenetic tree was generated using MEGA X. Bootstrap values from 1,000 repetitions are shown next to branches. The tree with the maximum log-likelihood is shown. Scale = substitutions per site. **(B)** Schematic locus of VviNPF2.1 and 2.2 on chromosome 6 with 3 other annotated genes. **(C)** The protein sequence alignment of VviNPF2.1, VviNPF2.2, AtNPF2.3 (At3g45680), and AtNPF6.3 (At1g12110). The proton coupling motif ExxER, and the key residues His-356 and Phe-511 for the NO_3_^–^ transporting feature of AtNPF6.3 are labeled. Colors represent amino acid similarity levels scored using Blosum62 score matrix (green: 100% similar; olive: 80–100% similar; orange: 60–80% similar; clear: < 60% similar). The table shows the similarity and identity between the proteins aligned. Protein alignments were generated using Clustal W (Larkin et al., 2007) using Geneious version 8.1.7 with the default settings.

Phylogenetically, VviNPF2.1 and VviNPF2.2 share a close relationship, and to *A. thaliana* NPF2.1–2.7 (Figure 1A). The two genes are adjacent on chromosome 6 in the grapevine reference genome (*V. vinifera* cultivar PN40024) (Jaillon et al., 2007) with no genes in between (Figure 1B). The promoter regions of *VviNPF2.1* and *VviNPF2.2* (approximately 1.2 kb upstream of the predicted start codon) are 92.2% identical. Protein alignment revealed that VviNPF2.1 and VviNPF2.2 have a high degree of homology as their amino acid sequences are 96.7% identical and 97.7% similar (Figure 1C).

### VviNPF2.1 and VviNPF2.2 Encode Plasma Membrane Localized Proteins and Are Highly Expressed in Grapevine Roots and Leaves

**FIGURE 2:**
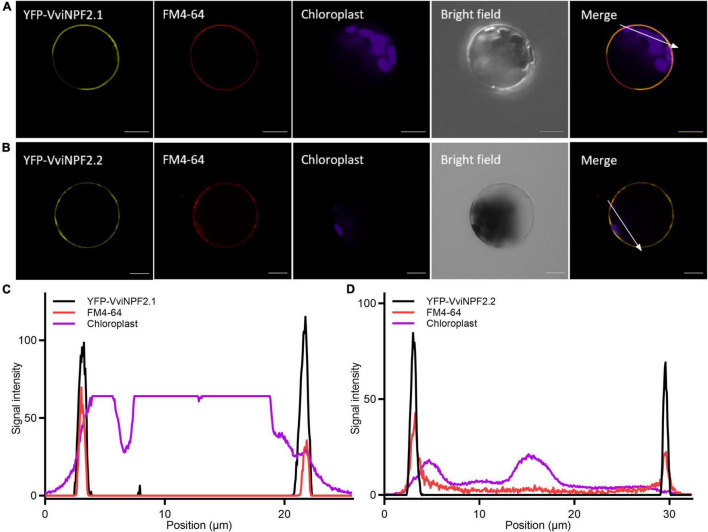
VviNPF2.1 and VviNPF2.2 localize to the plasma membrane in Arabidopsis mesophyll protoplasts. Confocal laser-scanning microscope images of **(A)** YFP-VviNPF2.1 co-localized with FM4-64, and **(B)** YFP-VviNPF2.2 co-localized with FM4-64. Whole protoplasts were imaged 16 h post-transfection with vectors encoding 35S:*EYFP-VviNPF2*. FM4-64 was applied to protoplasts 15 min before imaging. Scale bars = 10 μm. **(C,D)** Signal intensity profiles of YFP (black line), FM4-64 (red line), and chloroplast (magenta line) corresponding to the white arrow in the merged images in **(A,B)**. *X*-axis indicates the distance from the start to the end of the white arrow. Overlapping peaks indicate signal co-localization.

To investigate subcellular localization, amino (N-) terminal yellow fluorescent protein (YFP) fusions of VviNPF2.1 and VviNPF2.1 were transiently expressed in Arabidopsis (Col-0) mesophyll protoplasts. The N-terminal fusion proteins produced YFP signals that co-localized with the dye FM4-64, which after short periods predominantly stains the plasma membrane (Figure 2). VviNPF2.2 with C-terminal YFP was also localized to the plasma membrane (Supplementary Figure 1). Localization on the plasma membrane is consistent with most characterized plant NPF proteins to date (Corratgé-Faillie and Lacombe, 2017), and indicates that the grapevine NPF proteins could mediate substrate fluxes in or out of the cytoplasm, rather than the vacuole or other organelles.

**FIGURE 3:**
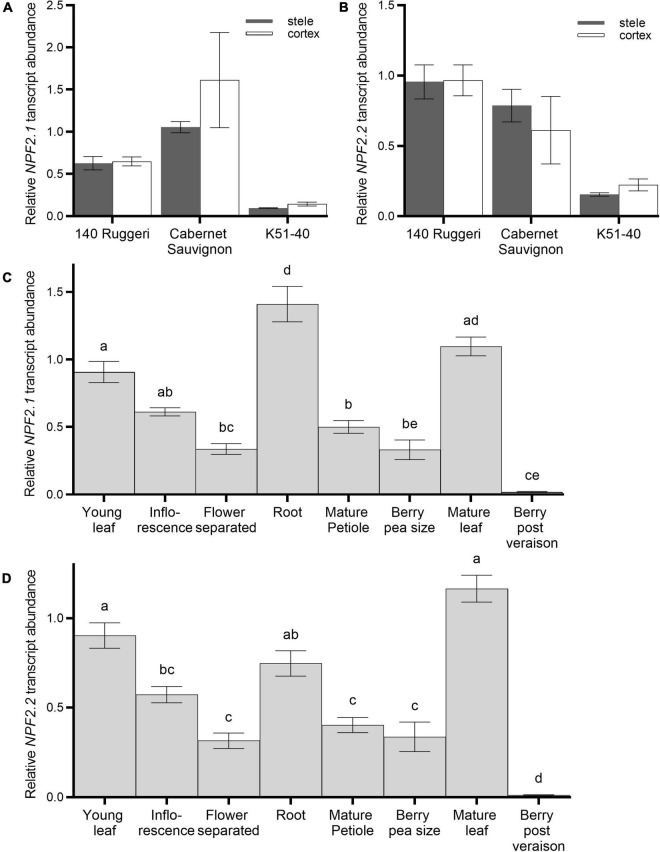
Expression patterns of *VviNPF2.1* and *VviNPF2.2* in grapevines. **(A,B)** Transcript abundance of **(A)** *VviNPF2.1* and **(B)** *VviNPF2.2* in root stelar-enriched (gray bars) or root cortex/epidermis-enriched (white bars) fractions of hydroponically grown roots harvested from rooted leaves of 140 Ruggeri, Cabernet Sauvignon and K51-40. There were no statistically significant differences between expression levels in stelar- and cortex-enriched fractions in all groups (*p* < 0.05, Student’s *t*-test). Data are the mean normalized expression level relative to the Cabernet Sauvignon cortical replicate 1 ± SE (*n* = 3). **(C,D)** Relative transcript abundance of **(C)** *VviNPF2.1* and **(D)** *VviNPF2.2* in different tissue types of Cabernet Sauvignon. Cabernet Sauvignon plants were propagated from hardwood cuttings in pots and tissue samples were collected during the growing season. Each sample contains tissues harvested from 3 individual plants. Transcript abundance of each gene is relative to the abundance in young leaf sample 1. Different letters denote statistically significant differences in the mean (one-way ANOVA with Tukey’s multiple comparisons test, *p* < 0.05). Data are mean ± SE (*n* = 3).

Gene expression patterns of *VviNPF2.1 and VviNPF2.2* were investigated using RT-qPCR. In roots, the expression patterns were probed in dissected fractions. In fractions enriched in epidermal/cortical cells, or enriched in stelar cells, neither *VviNPF2.1* nor *VviNPF2.2* were differentially expressed in three grapevine cultivars (140 Ruggeri, Cabernet Sauvignon and K51-40) (Figures 3A,B). To investigate other cell types, Cabernet Sauvignon hardwood cuttings were propagated in pots and RT-qPCR gene expression analyses were performed on various tissue types harvested during the growing season. Both *VviNPF2.1* and *VviNPF2.2* were most highly expressed in the root, young leaf, and mature leaf samples, and their expression levels in post-veraison berries were very low (Figures 3C,D). Similar patterns were also observed when the Grapevine Gene Expression Atlas (Fasoli et al., 2012) was mined (Supplementary Figure 2). These results indicate that both *VviNPF2.1* and *VviNPF2.2* were relatively highly expressed in grapevine roots and leaves.

### Expression of Both *VviNPF2.1* and *VviNPF2.2* in Grapevine Roots Was Downregulated by Post-starvation High [NO_3_^–^] Resupply

**FIGURE 4:**
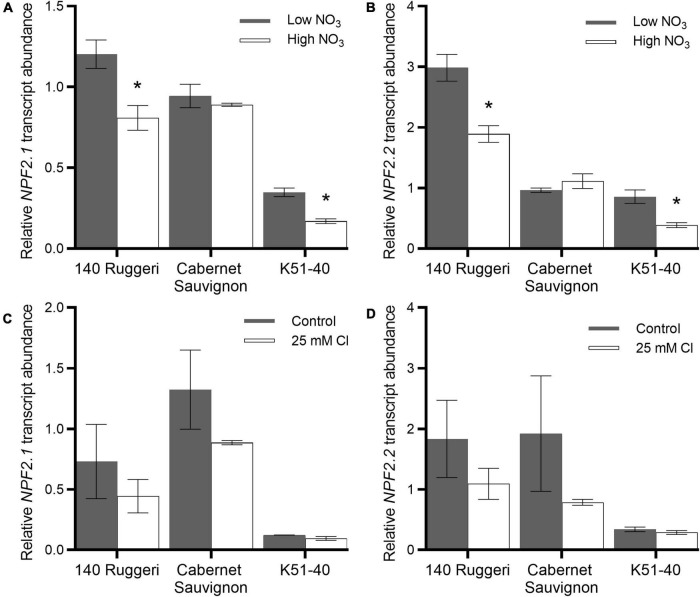
Effects of NO_3_^–^ and Cl^–^ treatments on root expression of *VviNPF2.1* and *VviNPF2.2*. Expression levels of *VviNPF2.1* and *VviNPF2.2* in roots of grapevine rootstock cuttings are downregulated by post-starvation high [NO_3_^–^] resupply, but not affected by Cl^–^ treatment. **(A,B)** Relative **(A)** *VviNPF2.1* and **(B)** *VviNPF2.2* transcript abundance in grapevine roots in response to the low NO_3_^–^ control condition (0.8 mM, gray bars) or high NO_3_^–^ resupply post-starvation (12 mM, white bars). **(C,D)** Relative **(C)** *VviNPF2.1* and **(D)** *VviNPF2.2* gene expression levels in grapevine roots in response to control (gray bars) or 25 mM Cl^–^ stress (white bars). Asterisks indicate a significant difference between anion treatments (*p* < 0.05, Student’s *t*-test). Data are mean ± SE (*n* = 3 replicates) and presented relative to Cabernet Sauvignon control sample 1.

To further investigate putative VviNPF2 function, expression levels of *VviNPF2.1* and *VviNPF2.2* in grapevine roots in response to different [NO_3_^–^] and [Cl^–^] treatments were analyzed. For NO_3_^–^ treatments, three grapevine cultivars—140 Ruggeri, Cabernet Sauvignon, and K51-40—were propagated from green cuttings and starved of NO_3_^–^ by growing in a low NO_3_^–^ medium (0.8 mM total NO_3_^–^) for 2 weeks. The plants were then supplied with either low NO_3_^–^ (0.8 mM, equivalent to the NO_3_^–^ starvation condition) or high NO_3_^–^ (12 mM) solutions (Cochetel et al., 2017). The transcript abundance of *VviNPF2.1* and *VviNPF2.2* at 24 h post NO_3_^–^ treatment were both higher in the roots supplied with continually low [NO_3_^–^] than in the roots resupplied with high [NO_3_^–^] in 140 Ruggeri and K51-40 (Figures 4A,B).

For Cl^–^ treatments, rooted leaves of 140 Ruggeri, Cabernet Sauvignon, and K51-40 were grown in hydroponics and treated with either control or 25 mM Cl^–^ nutrient solutions (Henderson et al., 2014). RT-qPCR results showed that the transcript abundance in roots was not regulated by 25 mM [Cl^–^] stress (Figures 4C,D), with no significant differences being detected for *VviNPF2.1* and *VviNPF2.2* following Cl^–^ treatments. Collectively, these data suggest that the *NPF* transcripts respond to NO_3_^–^ but not Cl^–^.

### The Cl^–^ and NO_3_^–^ Transport Activities of VviNPF2.1 and VviNPF2.2 Could Not Be Confirmed in the *Xenopus* Oocyte System

To test if VviNPF2.1 and VviNPF2.2 could transport Cl^–^ and/or NO_3_^–^, we expressed them in *Xenopus* oocytes and incubated the oocytes in uptake buffers containing ^36^Cl^–^ or ^15^NO_3_^–^, then tested the isotope content in the oocytes after the uptake period. The Cl^–^ and/or NO_3_^–^ transporter *Torpedo* CLC-0 (Bergsdorf et al., 2009) was used as a positive control, and the water-injected oocytes were used as negative controls. The results show that the *CLC-0*-expressing oocytes had higher ^36^Cl^–^ content than the negative controls after the uptake period, but the *VviNPF2.1*- and *VviNPF2.2*-expressing oocytes had lower ^36^Cl^–^ counts compared to the water-injected oocytes (Supplementary Figure 3A). The ^15^NO_3_^–^ uptake result was similar, except that the ^15^NO_3_^–^ content in *VviNPF2.2*-expressing oocytes was not statistically different from that of the negative controls (Supplementary Figure 3B). To test if these uptake results were due to the anion efflux through VviNPF2.1 and VviNPF2.2, we injected the oocytes with ^36^Cl^–^ or ^15^NO_3_^–^ and tested the isotope content in the oocytes after a period of incubation in the efflux buffer. In this experiment, the positive control oocytes had significant reductions in ^36^Cl^–^ and ^15^NO_3_^–^, but the *VviNPF2.1*- and *VviNPF2.2*-expressing oocytes were not statistically different from the negative controls (Supplementary Figures 3C,D). We also attempted to test if VviNPF2.1 and VviNPF2.2 could alter the nominal [Cl^–^] in the oocytes. The oocytes were incubated in the same buffer for 2 days, then rinsed and dissolved in acid to test the [Cl^–^]. The Cl^–^ transporter *CLC-0*-expressing oocytes had lower [Cl^–^] than the negative controls, but the expression of *VviNPF*s was not able to significantly alter the oocyte [Cl^–^] (Supplementary Figure 3E).

### Expression of *VviNPF2.2* in Arabidopsis Root Epidermis and Cortex Affects Shoot [Cl^–^] and Xylem Sap [NO_3_^–^]

**FIGURE 5:**
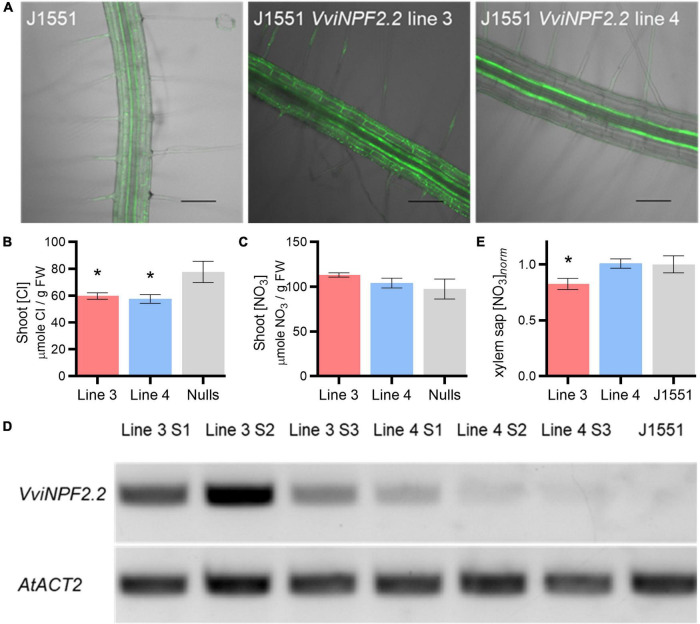
*VviNPF2.2* expression in Arabidopsis root epidermis, cortex and endodermis reduced shoot [Cl^–^] post-NaCl treatment. **(A)** Enhancer trap line J1551 mGFP5-ER expression pattern showing root vasculature specific transgene expression. Scale bars = 100 μm. **(B,C)** Arabidopsis J1551 *VviNPF2.2* expression lines 3 and 4 have **(B)** lower shoot [Cl^–^] than the null segregants (nulls) and **(C)** similar shoot [NO_3_^–^] compared to the nulls, after the 75 mM NaCl treatment. Data are means ± SE (*VviNPF2.2* expression lines, *n* = 21–22 samples; nulls, *n* = 12 samples). **(D)** Roots of Arabidopsis *VviNPF2.2* expression line 4 show lower transgene expression levels than those of line 3. Semi-quantitative RT-PCR shows *VviNPF2.2* and *AtACT2* (housekeeping gene) PCR fragments amplified from root cDNA of the line 3 samples 1–3 (S1–S3), the line 4 samples 1–3 (S1–S3), and a non-transformed J1551. **(E)** Line 3 has lower xylem sap [NO_3_^–^] than the non-transformed J1551. Data are the combination of two batches of plants and the xylem sap [NO_3_^–^] was normalized to the mean [NO_3_^–^] of J1551. Data are means ± SE (*VviNPF2.2* expression lines, *n* = 30–31 samples; J1551, *n* = 19 samples). Asterisks indicate statistically significant differences between the transgenic line and the control lines (*p* < 0.05, Student’s *t*-test).

As a plant system is likely to be more suitable for the functional characterization of VviNPF2s, we expressed one of the genes in Arabidopsis. *VviNPF2.2* was selected due to its higher expression in the strong Cl^–^ -excluder 140 Ruggeri compared to the poor excluder K51-40 (Supplementary Table 1; Henderson et al., 2014). A previous study suggested that PM-localized AtNPF2.5 could reduce shoot Cl^–^ accumulation *via* efflux from the root cortex (Li et al., 2017a). Therefore, Arabidopsis enhancer trap line J1551 was used; in this line, root cortex and epidermis-specific transgene expression is activated by binding of the transcription activator protein GAL4-VP16 to the *UAS* promoter (Plett, 2008; Plett et al., 2010). mGFP5-ER signals were observed in epidermis, cortex, and endodermis, confirming the cell-type-specific gene expression (Figure 5A). Two hydroponically grown J1551:*VviNPF2.2* independent lines (lines 3 and 4) were treated with 75 mM NaCl for 5 days and the shoots were analyzed for [Cl^–^] and [NO_3_^–^]. J1551:*VviNPF2.2* expression lines showed lower shoot [Cl^–^] compared to null segregants after NaCl treatment (Figure 5B), while shoot [NO_3_^–^] of each genotype was similar (Figure 5C). Expression of *VviNPF2.2* in line 3 and line 4 were assessed by quantitative RT-PCR from three replicates, relative to *AtACT2*. The results showed that *VviNPF2.2* was significantly more highly expressed in line 3 than in line 4 (Figure 5D and Supplementary Figure 4).

Xylem sap was collected from J1551:*VviNPF2* lines grown in standard basal nutrient solution (BNS), to determine whether *VviNPF2.2* expression affects xylem sap [NO_3_^–^] under normal conditions. The xylem sap [NO_3_^–^] of line 3, which had higher root *VviNPF2.2* expression, was significantly reduced compared to the J1551 control plants (Figure 5E). Line 4 with lower *VviNPF2.2* expression had the same xylem [NO_3_^–^] level as the control plants (Figure 5E).

## Discussion

Plasma membrane localization of VviNPF2.1 and VviNPF2.2 (Figure 2 and Supplementary Figure 1), suggests they facilitate solute fluxes to and from the cell cytoplasm. A greater abundance of *VviNPF2.1* and *VviNPF2.2* in the Cl^–^-excluding rootstock 140 Ruggeri compared to the Cl^–^-includer K51-40 (Figures 2, 3 and Supplementary Table 1) indicates that VviNPF2.1 and VviNPF2.2 could function in Cl^–^ transport. This would be consistent with the absence of key residues His-356 and Phe-511 required for NO_3_^–^ selectivity of AtNPF6.3 (Parker and Newstead, 2014; Sun et al., 2014). Cl^–^ permeability of VviNPF2.1 and 2.2 would also align with substrates of orthologous proteins from Arabidopsis, Maize, and Medicago (Li et al., 2016; Wen et al., 2017; Xiao et al., 2021). The transport activity of VviNPF2.1 and VviNPF2.2 is expected to be a passive efflux because both proteins lack the ExxER/K proton coupling motif present within the H^+^ symporting NPFs (Figure 1C; Jørgensen et al., 2015). However, amino acid sequence alignment showed that another known NO_3_^–^ effluxer AtNPF2.3 (Taochy et al., 2015) also lacked the ExxER proton coupling motif and the equivalent His-356 and Phe-511 residues of AtNPF6.3 (Figure 1C). Due to the comparatively low similarity between AtNPF6.3 and NPF2 proteins (27.7–32.8%), the AtNPF6.3 sequence alone might not reliably predict NPF2 substrates.

VviNPF2.1 and VviNPF2.2 expression in grapevines was not regulated by external Cl^–^ (Figure 4). Neither the RT-qPCR analyses on the 25 mM Cl^–^ -treated grapevine roots (Figures 4C,D) nor an additional 100 mM NaCl treatment of Cabernet Sauvignon roots (Supplementary Figure 5) showed any statistically significant differences in *VviNPF2* expression between control and salt treatment. This agrees with previous microarray gene expression analyses of grapevine roots (Henderson et al., 2014; Supplementary Table 1). Conversely, expression of *VviNPF2.1* and *VviNPF2.2* was downregulated by post-starvation high [NO_3_^–^] resupply in whole roots of 140 Ruggeri and K51-40 (Figures 4A,B). This indicates a possibility that VviNPF2.1 and VviNPF2.2 might function in NO_3_^–^ fluxes, though does not exclude permeability to Cl^–^.

We observed that the expression patterns of *VviNPF2.1* and *VviNPF2.2* in all experiments were similar. *VviNPF2.1* and *VviNPF2.2* are adjacent to one another on chromosome 6; according to the amino acid and nucleotide sequence analysis, *VviNPF2.1* and *VviNPF2.2* are highly similar (Figure 1) (as are their promoter regions, alignment not shown). This is likely to occur due to a gene duplication during evolution. Gene duplications are considered important evolutionary events which create chances for the emergence of new genes with new functions or with more specific functions (reviewed in Taylor and Raes, 2004). Considering the similarities between the sequences, the tissue expression patterns, and the expression responses to NO_3_^–^ and Cl^–^ of *VviNPF2.1* and *VviNPF2.2*, it is possible that they have not yet evolved to the stage at which expression differences emerge.

While assays to determine the Cl^–^ and NO_3_^–^ permeabilities of VviNPF2.1 and VviNPF2.2 using *Xenopus* oocytes were inconclusive, we observed in anion isotope uptake experiments that *VviNPF2.1-* and *VviNPF2.2*-expressing oocytes had lower ^36^Cl^–^ and/or ^15^NO_3_^–^ tracer levels than the negative controls. The isotope efflux results, however, did not support the possibility that the lower uptake was due to anion efflux through the VviNPFs (Supplementary Figures 3A–D). It has been previously found that the NO_3_^–^ transport of AtNPF2.3 could not be observed in the *Xenopus* oocyte system using ^15^NO_3_^–^ tracer, and the possibility of *Xenopus* oocytes not being a suitable system for the functional characterization of some NPF proteins has been discussed (Taochy et al., 2015). Regarding the observation that the *VviNPF2.1*-expressing oocytes had lower δ^15^N content in the ^15^NO_3_^–^ tracer uptake experiment (Supplementary Figure 3B), similar data were found in a previous ^15^NO_3_^–^ tracer uptake experiment performed by Léran et al. (2015). In their study, some of the *NPF*-expressing oocytes displayed lower relative ^15^N accumulation than that of the negative control oocytes, but the reason for this remains unknown. Consequently, similar to the conclusion of Taochy et al. (2015), we speculate that the *Xenopus* expression system might not be suitable for the functional characterization of some NPFs, including VviNPF2.1 and VviNPF2.2. However, it is possible that the VviNPFs failed to express in the oocytes, or were not directed to the plasma membrane. Future studies into grapevine NPF proteins could use a fluorescence protein tag to confirm the expression of VviNPFs on the plasma membrane of oocytes; or instead of the oocytes, use the *Lactococcus lactis* expression system that was used to successfully characterize AtNPF2.3 (Taochy et al., 2015). Future studies could also investigate the effect of different pH conditions on VviNPF function, or whether co-expression of an interacting partner protein is required for them to function correctly in oocytes.

Although we were unable to determine if VviNPF2.1 and VviNPF2.2 could transport the two anions in *Xenopus* oocytes, we were able to observe altered Cl^–^ and NO_3_^–^ accumulation in VviNPF2.2-expressing Arabidopsis plants. After applying salt stress to the root cortex and epidermis-specific *VviNPF2.2*-expressing J1551 (lines 3 and 4), we found that both lines had reduced shoot [Cl^–^] compared to the null segregant controls (Figure 5B), while the shoot [NO_3_^–^] of all genotypes was similar. This suggests that VviNPF2.2 may function in Cl^–^ efflux from the root cortex and epidermis to the external media, hence, reducing the amount of symplastic Cl^–^ available for translocation to shoots. However, unlike Cl^–^, NO_3_^–^ is assimilated by the plants. We suspect that due to nitrate assimilation, the shoot [NO_3_^–^] may not be a good representation of root-to-shoot NO_3_^–^ translocation. Therefore, we also used the same Arabidopsis *VviNPF2.2* expression lines for xylem sap [NO_3_^–^] measurements, so that the translocation could be more directly measured. Results showed that line 3 had significantly lower xylem sap [NO_3_^–^] than the control J1551 plants (Figure 5E), which suggests that VviNPF2.2 may also function in NO_3_^–^ efflux and could lead to a reduction of symplastic NO_3_^–^ through the leakage of NO_3_^–^ out of the roots. However, line 4 did not show [NO_3_^–^] differences compared to the controls (Figure 5E). It is important to note that line 3 and line 4 had very different *VviNPF2.2* expression levels; line 4 had significantly lower *VviNPF2.2* expression levels than line 3 (Figure 5D). We speculate that although high expression of *VviNPF2.2* could lead to significant leakage of NO_3_^–^, when the expression level is low, the NO_3_^–^ leakage could have been fully compensated by other Arabidopsis root nitrate transporters. Future studies could attempt to measure Cl^–^ and NO_3_^–^ efflux from the VviNPF-expressing J1551 roots to confirm whether reduced root-to-shoot anion transport is due to anion efflux from roots.

## Conclusion

In conclusion, VviNPF2.2 is likely to be a plasma membrane-localized passive Cl^–^ effluxer when expressed in Arabidopsis roots. The expression of *VviNPF2.2* in root epidermal and cortical cells could be beneficial to plants under salt stress, by promoting Cl^–^ efflux and reducing net shoot Cl^–^ accumulation.

## Data Availability Statement

The raw data supporting the conclusions of this article will be made available by the authors, without undue reservation.

## Ethics Statement

The animal study was reviewed and approved by the University of Adelaide Animal Research Ethics Committee.

## Author Contributions

YW performed all experiments, analyzed the data, and wrote the manuscript. SH assisted in the experiments. YW, SH, RW, and MG contributed to the design of the experiments. SH and MG conceived the project. SH, RW, and MG supervised the research and edited the manuscript. All authors read and approved the final manuscript.

## Conflict of Interest

The authors declare that the research was conducted in the absence of any commercial or financial relationships that could be construed as a potential conflict of interest.

## Publisher’s Note

All claims expressed in this article are solely those of the authors and do not necessarily represent those of their affiliated organizations, or those of the publisher, the editors and the reviewers. Any product that may be evaluated in this article, or claim that may be made by its manufacturer, is not guaranteed or endorsed by the publisher.
