# Supplementary material for: Root-Specific Expression of Vitis vinifera VviNPF2.2 Modulates Shoot Anion Concentration in Transgenic Arabidopsis
Source: Front Plant Sci. 2022 May 25;13:863971. doi: 10.3389/fpls.2022.863971 (PMC9174944; doi:10.3389/fpls.2022.863971)
Supplement: Supplementary file 1 [file Data_Sheet_1.docx]

Supplementary Material

## Supplementary Figures

**
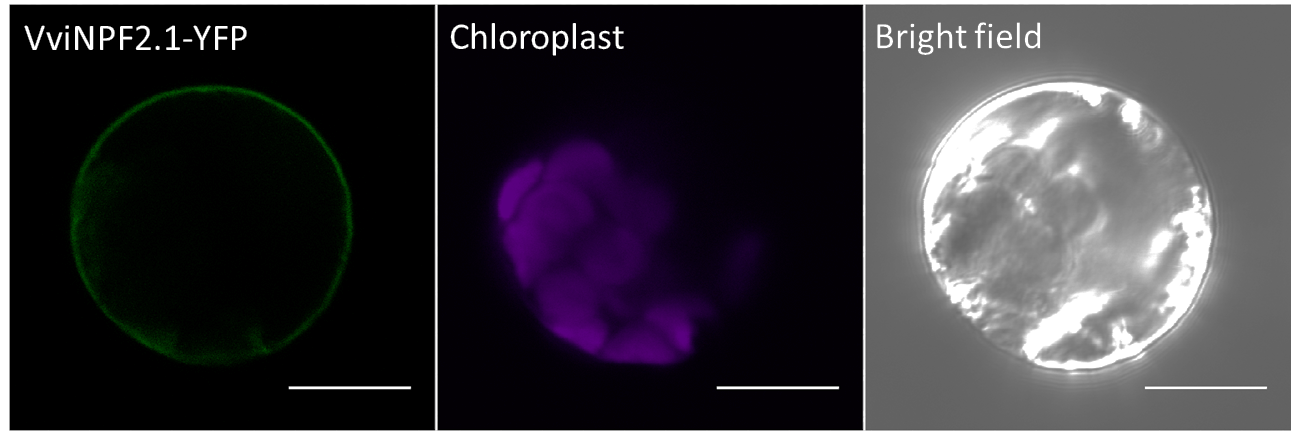
**

Supplementary Figure 1. **VviNPF2.1 with a C-terminus YFP tag localised to the plasma membrane in Arabidopsis mesophyll protoplasts.** Whole protoplasts were imaged 16 hours post transfection with vectors coding 35S:*VviNPF2.1-EYFP*. The left panel shows the YFP signal (green), the middle panel shows the chloroplasts (magenta), and the right panel is a bright field image of the protoplast. Scale bars = 10 μm.

**Supplementary Figure 2. *VviNPF2.1* and *VviNPF2.2* have similar expression patterns in the grapevine (*V. vinifera*, cv. Corvina).** Robust Multi-array Average **(**RMA) normalized signal intensities from the microarray gene expression study by Fasoli et al. (2012). Data presented in the heat map are the log2 mean transformed signal intensities of the 4 probes and 3 biological replicates. Abbreviations: L, latent; W, winter; S, swell; B, burst; AB, after-burst; Y, young; WD, well developed; FB, flower begins; F, flowering; FS, fruit set; S, senescing; PFS, post-fruit set; V, veraison; MR, mid-ripening; R, ripening; G, green; Stem-W, woody stem. The number after each sample name indicates the E-L growth stage (Dry et al., 2004) of the sample. The list of samples can be found in Fasoli et al. (2012) Supplemental Table 1.

**
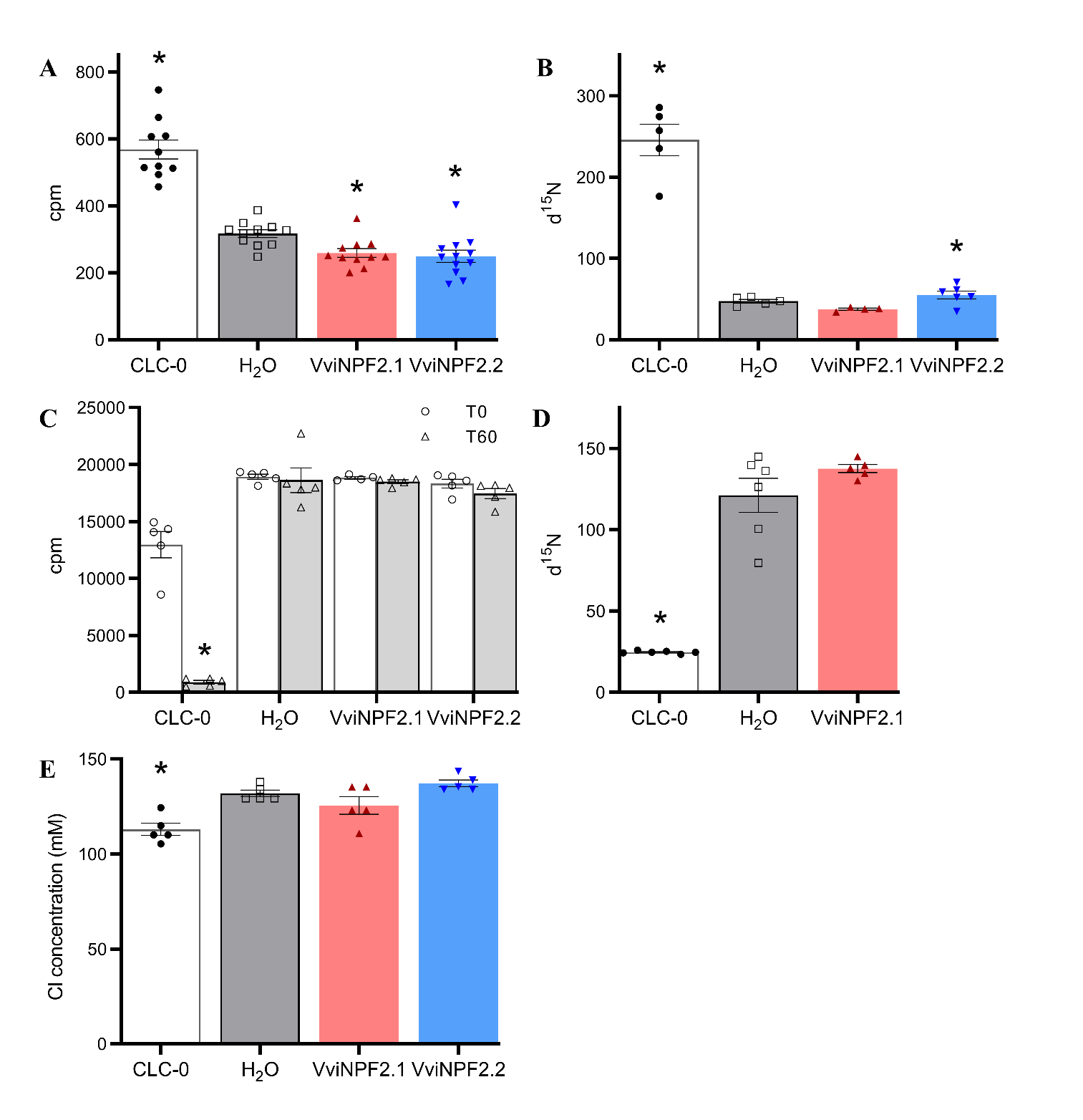
**

**Supplementary Figure 3. The anion transport activities of *VviNPF2.1*- and *VviNPF2.2*-expressing *Xenopus* oocytes.** **(A)** Oocyte isotope levels after incubation in influx solutions containing ^36^Cl^–^. Asterisks indicate statistically significant differences between cRNA-injected oocytes and in the water-injected controls (mean ± SE, *n* = 10-11 oocytes, P < 0.01, Student’s t-test). **(B)** Oocyte isotope levels after incubation in influx solutions containing ^15^NO_3_^–^. Asterisks indicate statistically significant difference between cRNA-injected oocytes and in the water-injected controls (mean ± SE, *n* = 4-5 samples, P < 0.05, Student’s t-test). **(C)** ^36^Cl^–^ injected oocyte isotope levels before (T0) and after (T60) incubation in efflux buffer. Asterisk indicates statistically significant differences between T0 and T60 (mean ± SE, *n* = 4-5 oocytes, P < 0.01, Student’s t-test). **(D)** ^15^NO_3_^–^ injected oocyte isotope levels after incubation in the efflux buffer for 60 min. Asterisk indicates statistically significant differences between the δ^15^N in the cRNA injected oocytes and in the water injected controls (mean ± SE, *n* = 5-6 samples, P < 0.01, Student’s t-test). **(E)** The [Cl^–^] in oocytes 2 days post incubation in Ca^2+^ Ringer’s solution. Asterisk indicates statistically significant differences between the [Cl^–^] in the cRNA injected oocytes and in the water injected controls (mean ± SE, *n* = 5 samples, P < 0.05, Student’s t-test).


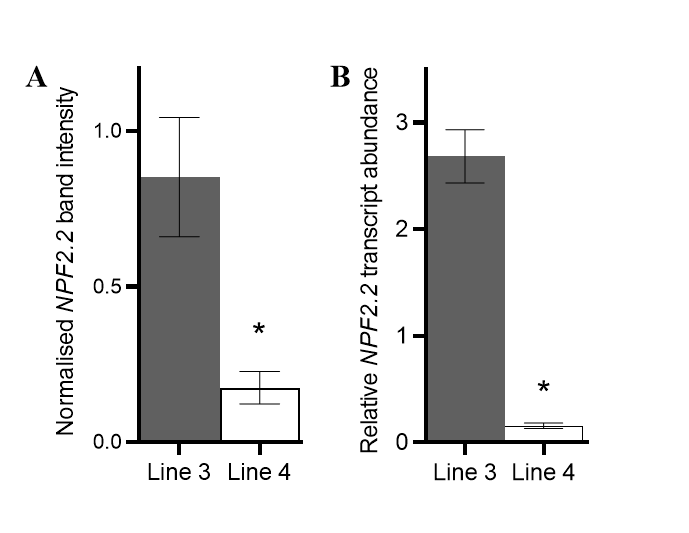


**Supplementary Figure 4.** Relative *VviNPF2.2* transcript abundance in Arabidopsis transgene expression lines tested by RT-qPCR is consistent with the semi qPCR results. **(A)** Normalised band intensities of *VviNPF2.2* in the semi qPCR gel image (Figure 5D). Intensity of each *VviNPF2.2* band is normalised to that of the corresponding *AtAct2* band. Asterisk indicates statistically significant difference between line 3 and line 4 (P < 0.05, Student’s t-test). Data are mean ± SE (*n* = 3). **(B)** Result of a 40-cycle-RT-qPCR shows that roots of Arabidopsis *VviNPF2.2* expression line 4 have significantly lower transgene expression levels than roots of line 3 (indicated by asterisk, P < 0.0001, Student’s t-test), which is consistent with the results in Figure 5D. Data are mean ± SE (*n* = 31 for line 3, *n* = 14 for line 4), and presented relative to line 3 sample 1.


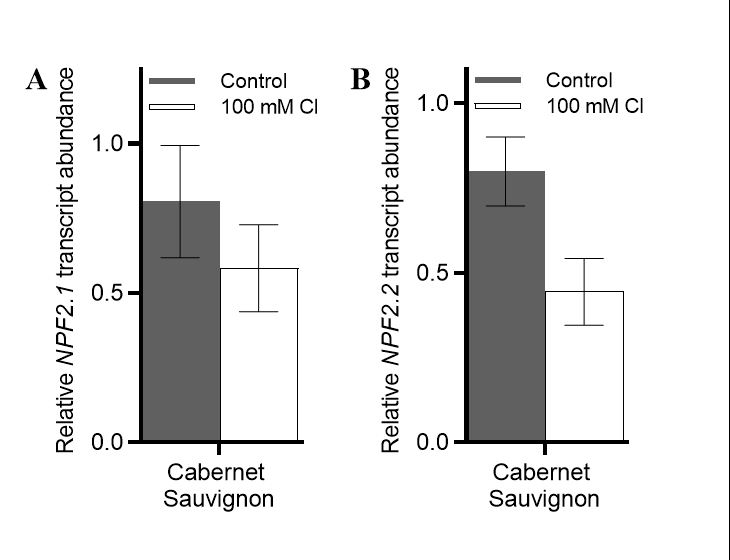


**Supplementary Figure 5. Expression levels of *VviNPF2.1* and *VviNPF2.2* in roots of Cabernet Sauvignon are not affected by the application of 100 mM Cl**^–^**.** **(A – B)** Relative (A) *VviNPF2.1* and (B) *VviNPF2.2* transcript abundance in Cabernet Sauvignon roots in response to control (grey bars) or 100 mM Cl^–^ stress (white bars). No statistically significant differences between the control and treatment groups are found (p < 0.05, Student’s t-test). Data are mean ± SE (*n* = 3 replicates), and presented relative to Cabernet Sauvignon control sample 1.

## Supplementary Table

**Supplementary Table 1. *VviNPF2.1* and *VviNPF2.2* expression data in a microarray gene expression study (Henderson et al., 2014).** The study shows that *VviNPF2.1* and *VviNPF2.2* are both more highly expressed in the roots of 140 Ruggeri (140R) than in K51-40, however expression was not significantly affected by the 50 mM Cl^−^ treatment in 140 Ruggeri (140R), Cabernet Sauvignon (CS) and K51-40 roots. Ctrl = control.

| **Genes** | **Control vs. Control** | | | **50 mM Cl vs. Control** | | | | | | | | |
| --- | --- | --- | --- | --- | --- | --- | --- | --- | --- | --- | --- | --- |
|  | 140R - K51-40 | | | 140R 50Cl - 140R Ctrl | | | CS 50Cl - CS Ctrl | | | K51-40 50Cl - K51-40 Ctrl | | |
|  | log(2) FC | adj.P.Val | B | log(2) FC | adj.P.Val | B | log(2) FC | adj.P.Val | B | log(2) FC | adj.P.Val | B |
| ***VviNPF2.1*** | 1.27 | 0.00 | 34.49 | 0.16 | 0.53 | -5.62 | -0.22 | 0.19 | -5.09 | 0.05 | 0.94 | -6.41 |
| ***VviNPF2.2*** | 1.61 | 0.00 | 66.55 | -0.01 | 0.99 | -6.24 | -0.21 | 0.11 | -4.52 | -0.40 | 0.00 | -0.44 |

The data (obtained with permission of Henderson et al. (2014)) is presented as log(2) gene expression fold change (FC) with adjusted p values and B values.

**Supplementary Table 2. Primers used in this study for molecular cloning and RT-qPCR analyses.**

| **Gene** | **Direction** | **Sequence (5'-3')** | **Technique** |
| --- | --- | --- | --- |
| *VviNPF2.1*/*VviNPF2.2* | Forward | ATGGCAGCCGCAGACCTGCAAA | Molecular cloning |
|  | Reverse | TCACTGATCGGACCCATTACTTG |  |
| *VviNPF2.1*/*VviNPF2.2* without stop codon | Forward | ATGGCAGCCGCAGACCTGCAAA |  |
|  | Reverse | CTGATCGGACCCATTACTTGTTC |  |
| *proVviNPF2.1* | Forward | GTTAGATCCACTTGGTTCATTTG |  |
|  | Reverse | TTGGTCTATGTTATTTGGTATATGAG |  |
| *proVviNPF2.2* | Forward | CACCCCTACCCATGACATTCCCA |  |
|  | Reverse | TTGGTCTATGTTATTTTGGTATATGAG |  |
| *VviNPF2.1* qPCR fragment | Forward | GCCAAATTCTCAATGCCCTT | RT-qPCR analysis |
|  | Reverse | CAGGCTGGTTCTGGAGGTG |  |
| *VviNPF2.2* qPCR fragment | Forward | ATCGCATCCTATGTCCATTATG |  |
|  | Reverse | CAGGCTGGTTCTGGAGGTG |  |
| *VviEF1-α* qPCR fragment | Forward | GAACTGGGTGCTTGATAGGC |  |
|  | Reverse | AACCAAAATATCCGGAGTAAAAGA |  |
| *VviTUA* qPCR fragment | Forward | CAAAACCAAACGGACTGTTCAAT |  |
|  | Reverse | GCACCTTAGCAAGATCACCC |  |
| *VviUBC* qPCR fragment | Forward | GTGGAGCCCTGCACTTACC |  |
|  | Reverse | GAGGGTCGTCAGGATTTGGA |  |

## References

Dry, P.R., Coombe, B.G., and Anderson, C.J. (2004). *Viticulture volume 1 – resources.* Adelaide: Winetitles.

Fasoli, M., Dal Santo, S., Zenoni, S., Tornielli, G.B., Farina, L., Zamboni, A., Porceddu, A., Venturini, L., Bicego, M., Murino, V., Ferrarini, A., Delledonne, M., and Pezzotti, M. (2012). The grapevine expression atlas reveals a deep transcriptome shift driving the entire plant into a maturation program. *Plant Cell* 24**,** 3489-3505.

Henderson, S.W., Baumann, U., Blackmore, D.H., Walker, A.R., Walker, R.R., and Gilliham, M. (2014). Shoot chloride exclusion and salt tolerance in grapevine is associated with differential ion transporter expression in roots. *BMC Plant Biology* 14**,** 273.
